# *GsMATE* encoding a multidrug and toxic compound extrusion transporter enhances aluminum tolerance in *Arabidopsis thaliana*

**DOI:** 10.1186/s12870-018-1397-z

**Published:** 2018-09-29

**Authors:** Qibin Ma, Rong Yi, Lu Li, Zhongyi Liang, Tingting Zeng, Yu Zhang, He Huang, Xiao Zhang, Xiangli Yin, Zhandong Cai, Yinghui Mu, Yanbo Cheng, Qiaoying Zeng, Xiuping Li, Hai Nian

**Affiliations:** 1 The State Key Laboratory for Conservation and Utilization of Subtropical Agro- bioresources, South China Agricultural University, Guangzhou, Guangdong 510642 People’s Republic of China; 2 The Key Laboratory of Plant Molecular Breeding of Guangdong Province, College of Agriculture, South China Agricultural University, Guangzhou, Guangdong 510642 People’s Republic of China; 3 The National Engineering Research Center of Plant Space Breeding, South China Agricultural University, Guangzhou, Guangdong 510642 People’s Republic of China; 4 The Experimental Teaching Center of Public Basic Courses, South China Agricultural University, Guangzhou, Guangdong 510642 People’s Republic of China; 5 The Guangdong Provincial Bioengineering Institute, Guangzhou, Guangdong 510316 People’s Republic of China; 6 The Guangdong AIB Polytechnic, Guangzhou, Guangdong 510316 People’s Republic of China

**Keywords:** *GsMATE*, Al tolerance, *Glycine soja*, *Arabidopsis thaliana*

## Abstract

**Background:**

Multidrug and toxic compound extrusion (MATE) transporters, which exist widely in plants, function as crucial regulators in plant resistance to aluminum (Al) toxicity by inducing citrate efflux. However, the functions of most MATE family members in soybean (*Glycine soja*) remain to be elucidated.

**Results:**

Expression pattern analysis showed that *GsMATE* was constitutively expressed in different soybean organs, with the highest level in root compared with those in stem, leaf and cotyledon. In addition, Al stress induced expression of *GsMATE* in soybean. Temporal analysis indicated that *GsMATE* expression was greatly enhanced by increasing concentrations of aluminum [Al^3+^] after short exposure, reaching the high levels detected in the BW69 (Al-resistant) and the JW81 (Al-sensitive) lines of *Glycine soja* of wild soybean at 6 h and 8 h, respectively. Furthermore, transient GsMATE expression in *Arabidopsis* protoplasts showed that GsMATE protein localized to the plasma membrane. Overexpression of *GsMATE* on an *Arabidopsis columbia*-0 (Col-0) background resulted in increased Al tolerance in transgenic plants. Analysis of hematoxylin staining showed that the roots of *GsMATE* transgenic lines were stained less intensely than those of the wild-type exposured to the same AlCl_3_ concentrations. Therefore, *GsMATE* enhanced the resistance of transgenic plants to Al toxicity by reducing Al accumulation in *Arabidopsis* roots.

**Conclusions:**

In summary, our results indicate that *GsMATE* is responsive to aluminum stress and may participate in the regulation of sensitivity to Al toxicity in *Arabidopsis*. In addition, the GsMATE protein is an Al-induced citrate transporter of the MATE family and exerts an essential role in Al tolerance in *Glycine soja*.

**Electronic supplementary material:**

The online version of this article (10.1186/s12870-018-1397-z) contains supplementary material, which is available to authorized users.

## Background

Acid soils (pH < 5.5), representing up to 50% of arable land, are widely distributed in developing countries in Africa, Asia, and South America [1–3]. Solubilized aluminum (Al), the most toxic trivalent cation (Al^3+^) which forms an Al complex in aluminosilicate clays (pH < 5.5), is a major limiting factor for plant growth and crop yield in acidic soils [4]. Al^3+^ toxicity primarily damages the root apex, causing significant reductions in plant growth and development by affecting the plasma membrane structure, inducing root cell death and inhibiting nutrient uptake [5, 6]. To date, two main types of Al-resistance mechanisms have been investigated in most plant species such as maize, wheat, and sorghum. The exclusion mechanism prevents Al from entering the root apex, while tolerance mechanisms detoxify and sequester Al in plants [1, 3]. Under Al stress, plants enhance their resistance to Al toxicity by root exudation of organic acids including malate, citrate and oxalate [7–11]. Multidrug and toxic compound extrusion (MATE) transporters have recently become the most categorized multidrug efflux transporter family. These proteins couple with substrate translocation across the plasma membrane with an electrochemical gradient of cations (such as H^+^ or Na^+^ ions) [12]. X-ray crystallography indicated a unique structural topology of the predicted 12 transmembrane (TM) helices in the MATE transporter that was distinct from that of other multidrug resistance transporters [13]. MATE transporters exist widely in bacteria, fungi, mammals and plants [14]. Many MATE genes, which encode proteins that induce citrate efflux in response to aluminum toxicity, have also been investigated and characterized in plants [1]. Using map-based cloning, SbMATE and HvAACT1 were the first MATE transporters shown to be involved in detoxification of Al in sorghum and barley [15, 16]. Since then, a number of MATE genes have been reported to be involved in the Al-induced secretion of citrate in herbaceous plants. These genes include *AtMATE* in *Arabidopsis* [6, 17], *TaMATE* in wheat [18], *ZmMATE1* in maize [19], *ScFRDL1* in rye [20], *HvAACT1* in wheat and barley [21], *SbMATE* in barley [22], *BoMATE* in *Arabidopsis* [23], *OsFRDL2* in rice [24], *FeMATE1* and *FeMATE2* in buckwheat [17], *BdMATE* in *Setaria viridis* [25], and *MtMATE66* in *Medicago truncatula* [26]. All these homologous genes encode MATE proteins that are required for external Al-resistance, and are primarily localized to the root epidermis cells [16, 17, 20, 23]. Some plant MATE transporters play diverse roles in iron homeostasis [26–32]; heavy metal [33], and toxin resistance [34]; vacuolar transport of nicotine [35–37]; chloride channels [38]; abscisic acid (ABA) efflux [39]; transport of secondary metabolites such as alkaloids, flavonoids, and anthocyanins [36, 39–42]; hypocotyl cell elongation [43]; export of hydroxycinnamic acid amides [44]; organ initiation [45]; regulation of lateral organ size and initiation rate [46]; plant growth and development [47]; the establishment of plant disease resistance [48]; and resistance to viruses [49], etc.

Previous studies have shown that the MATE proteins are a large family of multidrug efflux transporters in plants [12]. Many putative MATE transporters have been predicted and identified by genome-wide analysis and/or other methods in plants. These putative MATE transporters include 45 in *Oryza sativa* [50], 49 in maize [51], 56 in *Arabidopsis thaliana* [33, 50], 67 in tomato [7], 70 in *Medicago truncatula* [26, 52], 71 in *Populus* [1], 70 MATE genes in *Gossypium raimondii* and 68 MATE genes in *Gossypium arboretum* [53], and 117 in *Glycine max* [54]. Compared with other plant species, soybean has the largest MATE family with 117 putative MATE transporters predicted by genome-wide association analysis and RNA-seq Atlas of *Glycine max* [54]. However, few studies have been conducted on MATE transporters in soybean. To date, only two MATE transporters, GmFRD3a and GmFRD3b, have been reported to play a role in iron homeostasis in soybean [31, 55]. In the current study, *GsMATE* (accession number: BM732932.1) was cloned from the wild soybean root of the BW69 line (Al-resistant) of *Glycine Soja*, and the gene expression pattern was detected in response to Al treatment. The function of GsMATE was identified and characterized on subcellular localization and citrate transport activity along with phenotypic analysis of transgenic overexpression lines of *Arabidopsis*. We hypothesized that GsMATE would enhance Al tolerance in *Arabidopsis* via Al-induced secretion of citrate from the root.

## Methods

### Plant materials and growth conditions

The BW69 (Al-resistant) and the JW81 (Al-sensitive) lines of *Glycine soja* were used to clone *GsMATE* for investigation of *GsMATE* expression patterns in response to Al stress. The wild soybean seed coat was lacerated gently with a single-sided blade on the back of the hilum. Then, all seeds of wild soybean were grown under growth chamber (22–25 °C, 12-h/12-h light/dark cycle) as described previously by Zeng [56]. After germination, the seedlings were pre-cultured for 48 h in surface-sterilized vermiculite and transplanted into the nutrient solution (pH 5.8) after the spread cotyledons. After two days cultured at 22/25 °C with a 12-h/12-h light/dark cycle, the soybean seedlings were treated in aluminum solutions [56].

Ecotype Col-0 of *Arabidopsis* was used for *GsMATE* gene transformation. All the surface-sterilized *Arabidopsis* seeds (wild-type and transgenic lines) were cultivated on 1/2 Murashige and Skoog (MS) agar medium in darkness for 2 days at 4 °C. The *Arabidopsis* seedlings were then transferred to new 1/2 MS medium containing aluminum concentration gradients for continuous culture for several days at 22 °C under long-day conditions until all the samples were taken [57].

### GsMATE expression analysis

To analyze the tissue expression pattern of *GsMATE*, the BW69 and JW81 lines were germinated in sterilized vermiculite. The seedlings with open cotyledons were cultured in the nutrient solutions for four days. Samples of root, stem, leaf and cotyledon were taken from the seedlings (*n* = 10 plants per group), frozen in liquid nitrogen, and then stored at − 80 °C [56]. To analyze the influence of the Al concentration gradient on gene expression, the BW69 and JW81 lines were planted and germinated using the method described previously [56]. In brief, after pre-incubation for two days, the soybean seedlings were transferred and cultured in the solutions of 0, 25, 50, 75, and 100 μM AlCl_3_ (pH 4.3, 0.5 mM CaCl_2_), respectively; three replicates of 20 seedlings were prepared for each group. After treatment with aluminum solutions for 6 h, root tip samples (6 cm long) were obtained from the seedlings (*n* = 10 plants used per group). Samples were frozen in liquid nitrogen and then stored at − 80 °C [56]. To analyze the pattern of *GsMATE* expression in different root segments in response to Al toxicity, the soybean seedlings were prepared using the method described previously. Samples were obtained from seedling roots (sections 0–2, 2–4, and > 4 cm) after 6 h of treatment in Al solutions (*n* = 10 plants per group), frozen in liquid nitrogen and stored at − 80 °C for further analysis [56]. To analyze the temporal expression pattern of *GsMATE* in response to Al toxicity, two-day-old seedlings were cultured in a solution of 0.5 mM CaCl_2_ (pH 4.3) for 12 h. Root tip samples (6 cm long) were obtained from the seedlings after the time course treatments set as 0, 6 and 12 h, respectively. The other seedlings were then transferred to a solution of 50 μM AlCl_3_ (pH 4.3, 0.5 mM CaCl_2_) and cultured for 24 h (*n* = 20 seedlings per group). Root tip samples (6 cm long) were obtained from the seedlings treated after 2, 4, 6, 8, 12, and 24 h, respectively. All the samples were frozen in liquid nitrogen, and stored at − 80 °C for further analysis [56].

### Cloning of GsMATE

According to our previous analysis of the expression profiles of genes involved in acidic aluminum tolerance in BW69 and JW81 lines, one Al-induced *GsMATE* gene was identified by screening the database of the National Center for Biotechnology Information (NCBI) with the sequence information (unpublished data); the gene was assigned the accession number of BM732932.1. Specific primers (Additional file 1: Table S1) were designed to amplify the full-length sequence of *GsMATE* by RT-PCR using Super-Fidelity DNA polymerase (Phanta Max, Vazyme Biotech Co., Ltd.; Nanjing, China). Total RNA was extracted from the seedlings of BW69 under the treatment of 25 μM AlCl_3_ (pH 4.3, 0.5 mM CaCl_2_) using TRIzol reagent (Invitrogen). The cDNA was generated using 2 μg of total RNA by reverse transcription with SuperScript II reverse transcriptase (Invitrogen). Using cDNA of BW69 as a template, the reaction of RT-PCR was performed in a total volume of 20 μl according to a previously described method [57] followed by the amplification program: DNA denaturation at 94 °C for 30 s, annealing at 72 °C for 30 s, elongation at 72 °C for 1 min and 30 s (35 cycles). The *GsMATE* PCR product was isolated by 1% agarose gel electrophoresis (GenStar Kit, Genstar Development Company, Canada) and then inserted into the multiple cloning site of the pLB vector (Tiangen Rapid DNA Ligation Kit, Beijing, China). Clones of *E. coli* that were positively transformed into competent cells of DH5α strains with the GsMATE-pLB vector using the method of heat-shock were identified by PCR, enzyme digestion and sequencing (Sangon Biotech (Shanghai) Co., Ltd., China) to obtain the full cDNA sequence of *GsMATE*. The methods of PCR identification and enzyme digestion for the positive clones were previously described in detail [57, 58].

### Sequence analysis

Multiple alignments of sequences were carried out, and a homology tree was generated using DNAMAN software. The nucleotide and amino acid sequences were used to search for GsMATE and/or its homologous proteins using the BLAST network servers of the NCBI (https://blast.ncbi.nlm.nih.gov/Blast.cgi) and phytozome (https://phytozome.jgi.doe.gov/pz/portal.html) [57]. Prediction of the transmembrane topology of GsMATE protein was performed using the TMHMM Server website v. 2.0: http://www.cbs.dtu.dk/services/TMHMM/.

### Plasmid construction and transformation of GsMATE in Arabidopsis

The 1,503 bp GsMATE coding sequence (CDS) (Additional file 2) amplified from the GsMATE-pLB vector was inserted into the *Bam*HI and *Kpn*I sites of a pCAMBIA1301 vector with a β-glucuronidase (GUS) reporter to generate the pCAMBIA1301-GsMATE fusion construct under the control of the cauliflower mosaic virus 35S (CaMV 35S) promoter using specific primers (Additional file 1: Table S1). *Agrobacterium tumefaciens* GV3101 was then transformed with the pCAMBIA1301-GsMATE vector by electroporation, and full-flowering *Arabidopsis* plants were transformed using the floral dip method described previously [59]. All the *GsMATE* transgenic plants were obtained by hygromycin screening using 1/2 MS media.

### Localization of the GsMATE-GFP fusion protein

Localization of the GsMATE protein was performed using a previously described method [57]. Specific primers were used to amplify the full *GsMATE* CDS (Additional file 1: Table S1). The PCR product of the *GsMATE* CDS was subcloned into the *Bam*HI and *Kpn*I sites of the pYL322-d1 vector to generate a GsMATE-GFP fusion construct under the control of CaMV 35S promoter. The pYL322-d1-GsMATE-GFP construct was confirmed by sequencing and used for transient transformation of *Arabidopsis* protoplasts by heat-shock. Transformed *Arabidopsis* protoplasts were then observed under a confocal laser scanning microscope (Leica) to characterize GsMATE protein expression [57, 60].

### Quantitative real-time PCR

Total RNA was extracted from the seedlings of soybean or *Arabidopsis* using TRIzol reagent (Invitrogen) and treated with RNase-free DNase (Promega). Total RNA of 2 μg was used to generate cDNA by reverse transcription with SuperScript II reverse transcriptase (Invitrogen). The cDNA from each sample was then diluted to 4 and 8 ng/ml. Triplicate quantitative assays were performed on 1 ml of each cDNA dilution with the SYBR Green Master mix and the SsoFast EvaGreen Supermix Kit (BIO-RAD) on an ABI 7900 sequence detection system according to the manufacturers’ instructions. The data were normalized using the reference gene *β*-*tubulin*. The quantitative variation between the examined replicates was evaluated by the 2^–∆∆Ct^ method [57]. Details of the *GsMATE* and*β*-*tubulin* specific primers were listed in (Additional file 1: Table S1).

### Acidic aluminum treatment in transgenic Arabidopsis

For short-term AlCl_3_ treatment, T_3_ generation Arabidopsis seeds were sown in plastic petri dishes (10 cm diameter) filled with 1/2 MS phytagel medium (pH 5.8) and incubated at 4 °C in the dark for four days before transferred to a culture room (16-h light/8-h dark) at 22 °C for four days. Then, *Arabidopsis* seedlings were transferred to agarose medium containing AlCl_3_ (pH 4.5, 0.5 mM CaCl_2_) and cultured at 22 °C (16-h/8-h light/dark) for another two days. The seedlings were then exposed to AlCl_3_ (pH 4.5, 0.5 mM CaCl_2_) concentration gradients (0, 50, 100 and 200 μM), and main root measurements were carried out using a previously described method [61]. For long-term AlCl_3_ treatment, the Arabidopsis seedlings were cultured for 7 days or more to observe the Al-resistance phenotypes and/or measure the main root length [62].

Hematoxylin staining was used to further investigate the resistance of transgenic lines of *Arabidopsis* to Al toxicity [11]. *Arabidopsis* seedlings were prepared according to the previously described method. The seedlings were then transferred into AlCl_3_ solutions (pH 4.5, 0.5 mM CaCl_2_) for 6 h. After 30 mins rinse in ultrapure water, the roots of the *Arabidopsis* seedlings were stained with hematoxylin for 30 min. After another 30 mins rinse in ultrapure water, the *Arabidopsis* phenotypes were recorded [11].

### Statistical analysis

All data were represented as the mean ± SD of three biological replicates. The t-test at *p* = 0.05 was performed to identify significant differences between observation values using SPSS20 software [57].

## Results

### Cloning of GsMATE

Based on the gene expression profiles of Al-resistant *Glycine soja* (unpublished data), an aluminum-induced gene encoding a citrate transporter of MATE protein was cloned using the sequence of the *Glycine soja* BW69 line from the NCBI database under accession number BM732932.1. The MATE gene located on soybean chromosome 2 was then designated *GsMATE* (multidrug and toxin extrusion family protein of *Glycine soja*). The *GsMATE* gene was induced in more than twice expression level under the treatment of 25 μM AlCl_3_ (pH 4.3, 0.5 mM CaCl_2_) (data not shown). The full-length genomic *GsMATE* sequence included 13 exons and 12 introns, with a full-length cDNA of 1,955 bp (data not shown) and open reading frame (ORF) of 1,503 bp encoding 501 amino acids. The *GsMATE* sequence was deposited in the NCBI database under accession number BM732932.1 (Additional file 2: Table S2).

### Bioinformatics analysis of GsMATE

Phytozome quick search and NCBI BLAST analysis of the conservative domains revealed that GsMATE protein belongs to the MATE family of multidrug and toxin extrusion proteins (Additional file 3: Figure S1a). Prediction using TMHMM (Server v. 2.0) showed that GsMATE protein is a membrane protein with 10 transmembrane helices (domains) at the amino acid positions 145–167, 193–215, 225–247, 254–276, 291–313, 326–348, 368–390, 402–424, 434–456, and 463–485. Ring structures were formed using the transmembrane domains (Additional file 3: Figure S1b).

**Fig. 1:**
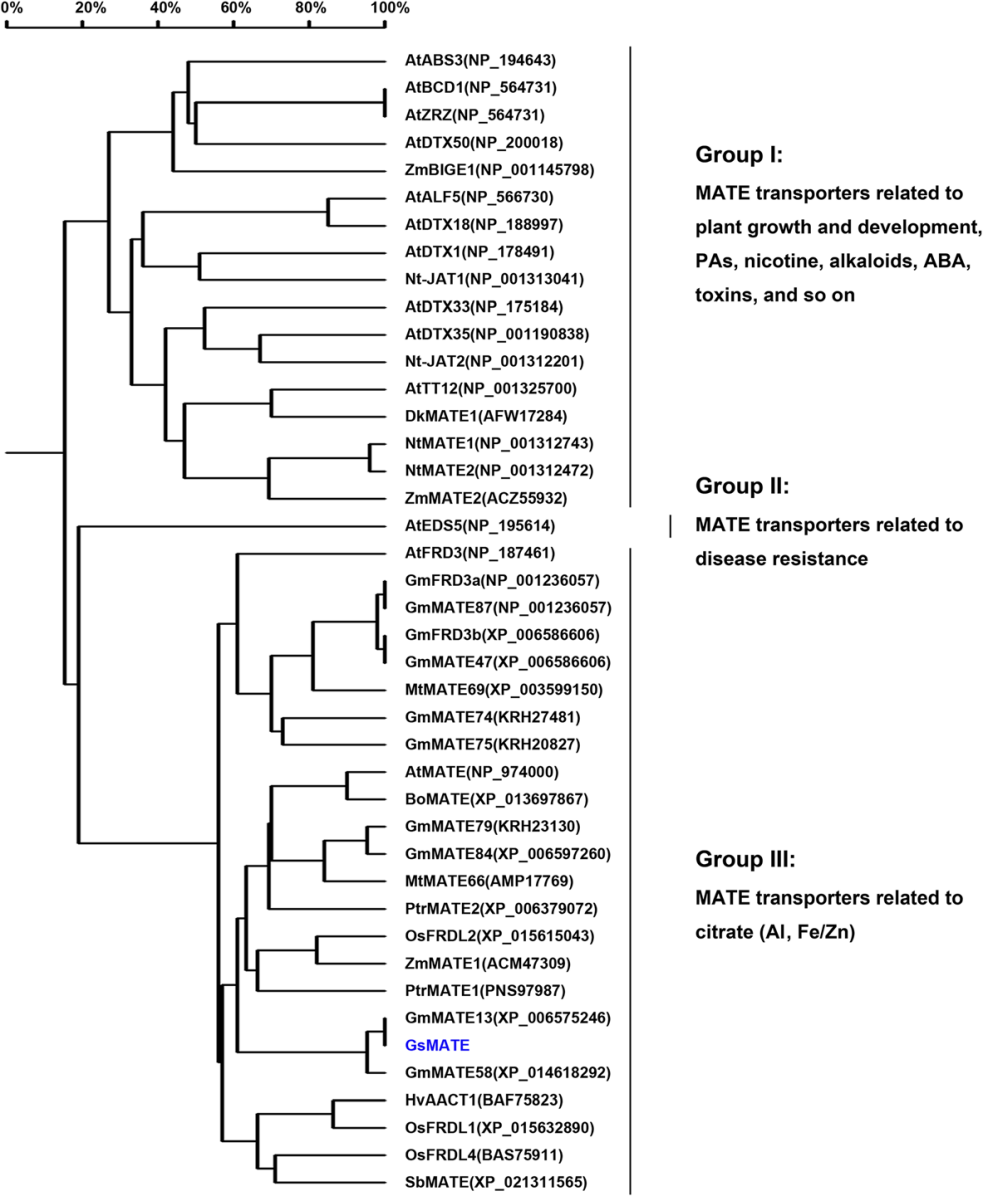
Homology analysis of GsMATE and other transmembrane proteins. All the available amino acid sequences and the accession numbers of MATE proteins were obtained from the NCBI databases (https://www.ncbi.nlm.nih.gov/). The MATE transporters have been characterized and their functions were identified except for GsMATE and the eight soybean MATE proteins [54]. The homology tree was produced using DNAMAN alignments. The accession numbers of the MATE proteins were shown in parentheses

Multiple sequence alignments and homology analysis showed differences among the members of MATE family in plants. As shown in Figs. 1, 42 MATE proteins were clustered into three groups. Group I comprised 17 MATE proteins with diverse potential functions, including regulation of organ initiation (AtZRZ and ZmBIGE1); compound transport and accumulation of alkaloids (NtMATE1), proanthocyanidins (DkMATE1), nicotine (Nt-JAT1, NtMATE1 and NtMATE2), chloride (AtDTX33 and AtDTX35); iron homeostasis (AtBCD); and responses to abiotic stresses such as drought (AtDTX50). Group II comprised only one member, AtEDS5, which is required for salicylic acid (SA) synthesis in pathogen-challenged plants [49]. There were 24 MATE proteins in group III performing major functions in Al-activated citrate transport or the regulation of iron homeostasis in plants (AtFRD3, GmFRD3a, GmFRD3b and OsFRDL1). At the amino acid level, GsMATE showed 56% similarity with other MATE transporters clustered in Group III, while the similarity among GsMATE, GmMATE13 and GmMATE58 exceeded 95% (Fig. 1). Therefore, the bioinformatics analysis indicated that GsMATE protein may function in Al tolerance.

### GsMATE expression pattern analysis

**Fig. 2:**
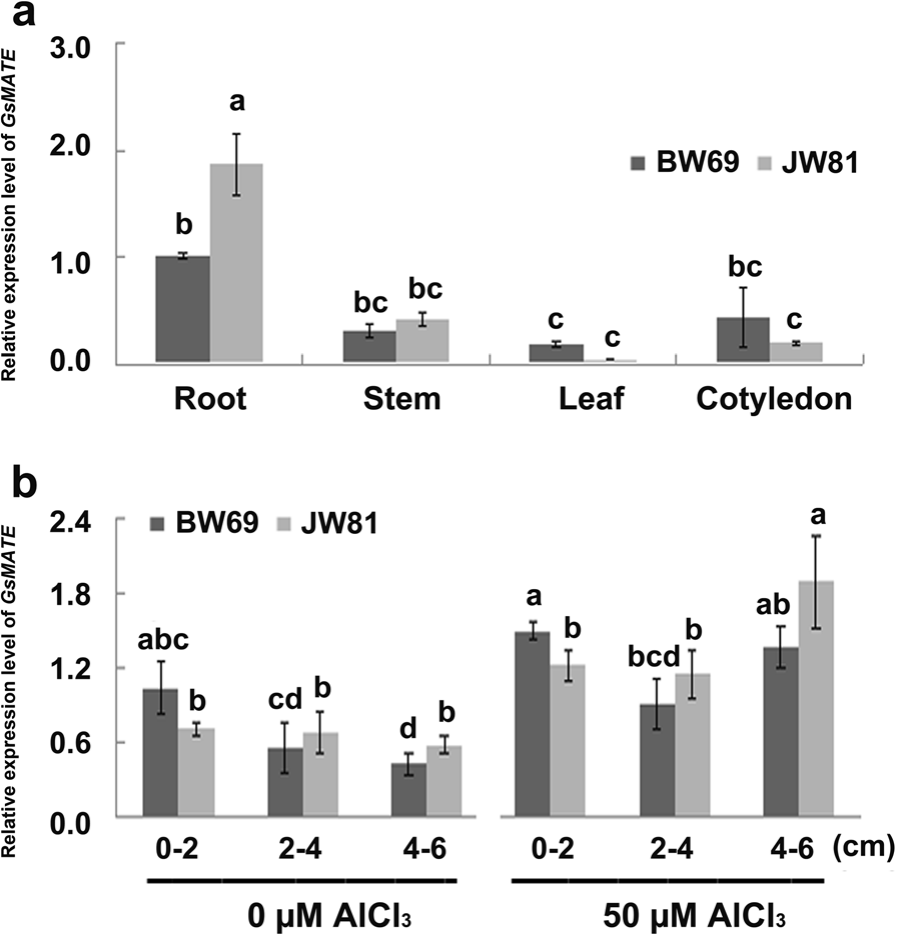
Tissue expression pattern of *GsMATE.* **a** Tissue expression pattern of *GsMATE.* **b** Expression of *GsMATE* in root sections. Tissue samples were obtained from the young BW69 and JW81 seedlings. Two days after germination, 20 seedlings were transferred to solutions of 0 μM and 50 μM AlCl_3_ (pH 4.3, 0.5 mM CaCl_2_). Samples were obtained from seedling roots (sections 0–2, 2–4, and > 4 cm) after 6 h of AlCl_3_ treatment. Data were represented as the mean ± SE of three biological replicates; the same letter on each column set indicates no significant difference and different letters indicated a statistically significant difference according to analysis of variance (t-test, *p* = 0.05). BW69, JW81: two lines of *Glycine soja*. Data were measured using Image J software, analyzed using SPSS20 and plotted using EXCEL2000

To investigate the tissue expression pattern of *GsMATE*, samples of young root, stem, leaf and cotyledon were taken from the BW69 and JW81 lines of *Glycine soja*. The analysis of quantitative real-time PCR (qRT-PCR) indicated that *GsMATE* is expressed constitutively in soybean with more than 3-fold expression levels in roots than those in stems and leaves (Fig. 2a). Further qRT-PCR analysis of *GsMATE* expression in different segments of the root measured from the tip showed that the highest and lowest expression under the check (0 μM AlCl_3_, pH 4.3, 0.5 mM CaCl_2_) in the 0–2 cm and 4–6 cm zones, respectively (Fig. 2b). However, the *GsMATE* gene was significantly up-regulated after Al treatment with almost twice expression levels in the root regions compared with those under the condition of the check (Fig. 2b). The *GsMATE* expression level at the root segment of the 4–6 cm zone of JW81 line was at least 3-fold expression level than that at the check treatment (Fig. 2b).

### Analysis of GsMATE responses to acidic aluminum

**Fig. 3:**
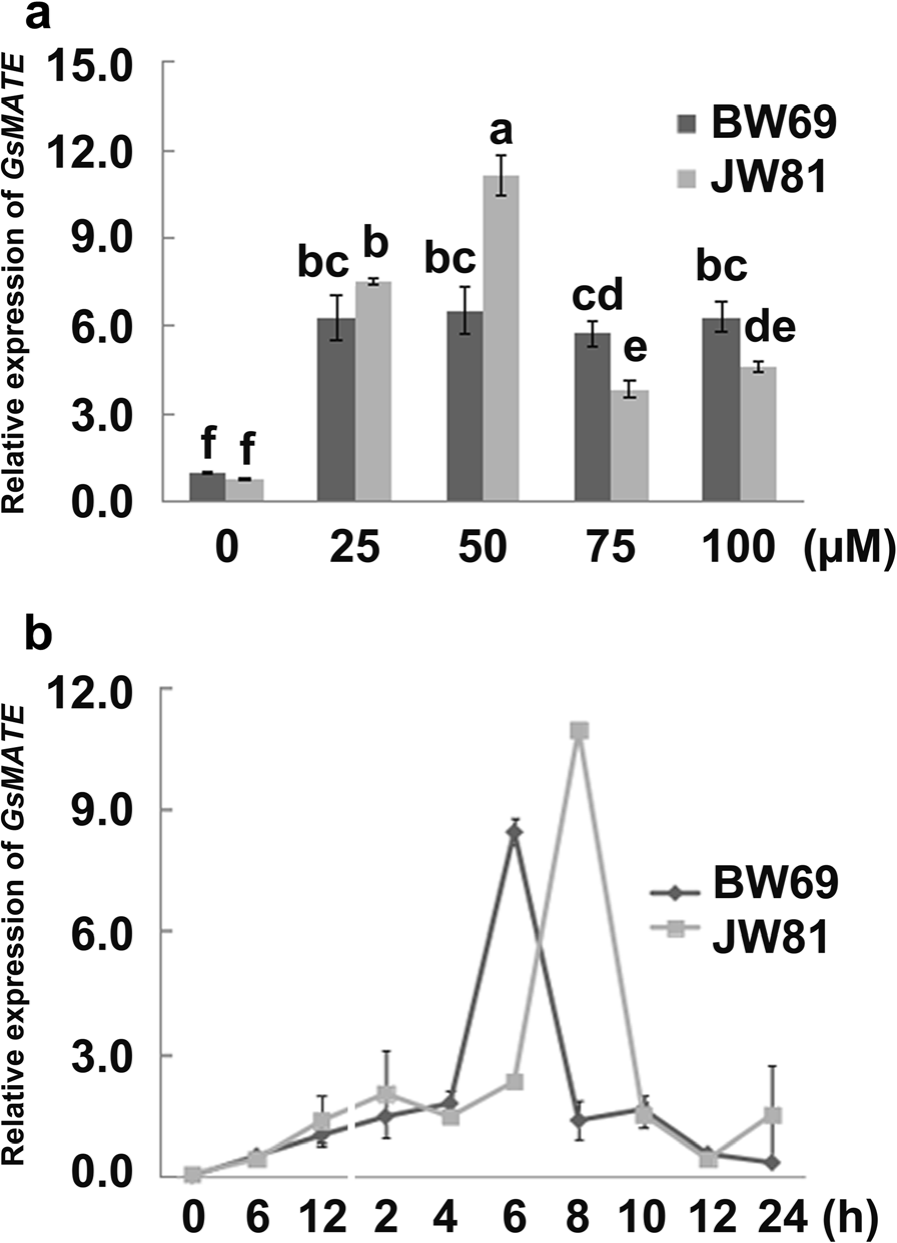
Pattern of *GsMATE* expression in response to acidic aluminum exposure. **a** Pattern of *GsMATE* expression under acidic aluminum exposure*.* **b** Temporal expression pattern of *GsMATE* under acidic aluminum exposure. Two days after germination, the seedlings were transferred to solutions of 0, 25, 50, 75, and 100 μM AlCl_3_ (pH 4.3, 0.5 mM CaCl_2_). After 6 h, root tip samples (6 cm long) were obtained from the seedlings for the analysis of the *GsMATE* expression patterns. To analyze the temporal expression pattern of *GsMATE*, seedlings (2 days after germination) were cultured in a solution of 0.5 mM CaCl_2_ (pH 4.3) for 24 h, and then transferred to the solution of 50 μM AlCl_3_ (pH 4.3, 0.5 mM CaCl_2_). Root tip samples (6 cm long) were obtained from the seedlings after the treatments of 2, 4, 6, 8, 12, and 24 h. Data were represented as the mean ± SE of three biological replicates; the same letter on each column set indicates no significant difference and different letters indicate a statistically significant difference according to analysis of variance (t-test, *p* = 0.05). BW69, JW81: two lines of *Glycine soja*. Data were analyzed using SPSS20, and plotted using EXCEL2000

*GsMATE* was significantly induced under exposure to the different Al concentration gradients, with 4-fold to 12-fold greater expression levels detected in the BW69 and JW81 wild soybean lines compared with those detected under control conditions (Fig. 3a). The *GsMATE* gene expressed highest level at the treatment of 50 μM AlCl_3_ with a significantly higher level up to 12-fold detected in the aluminum-sensitive JW81 line than those in the aluminum-resistant BW69 line. In contrast, at the higher concentrations of AlCl_3_ (75 and 100 μM), *GsMATE* expression in the aluminum-resistant BW69 line was significantly higher than that in the aluminum-sensitive JW81 line (Fig. 3a).

The temporal expression pattern of *GsMATE* in response to acidic aluminum exposure was analyzed over a period of 24 h (Fig. 3b). Although the expression level of *GsMATE* was low at pH 5.8, it was much higher than those detected at pH 4.3 with similar expression patterns observed in both the BW69 and JW81 lines. The Al-resistant BW69 line responded two-hours earlier to Al stress than the Al-sensitive JW81 line with the highest expression level of *GsMATE* up to 8.6-fold and 11-fold detected after treatment for 6 h and 8 h in the two lines, respectively (Fig. 3b).

### Subcellular localization of GsMATE

**Fig. 4:**
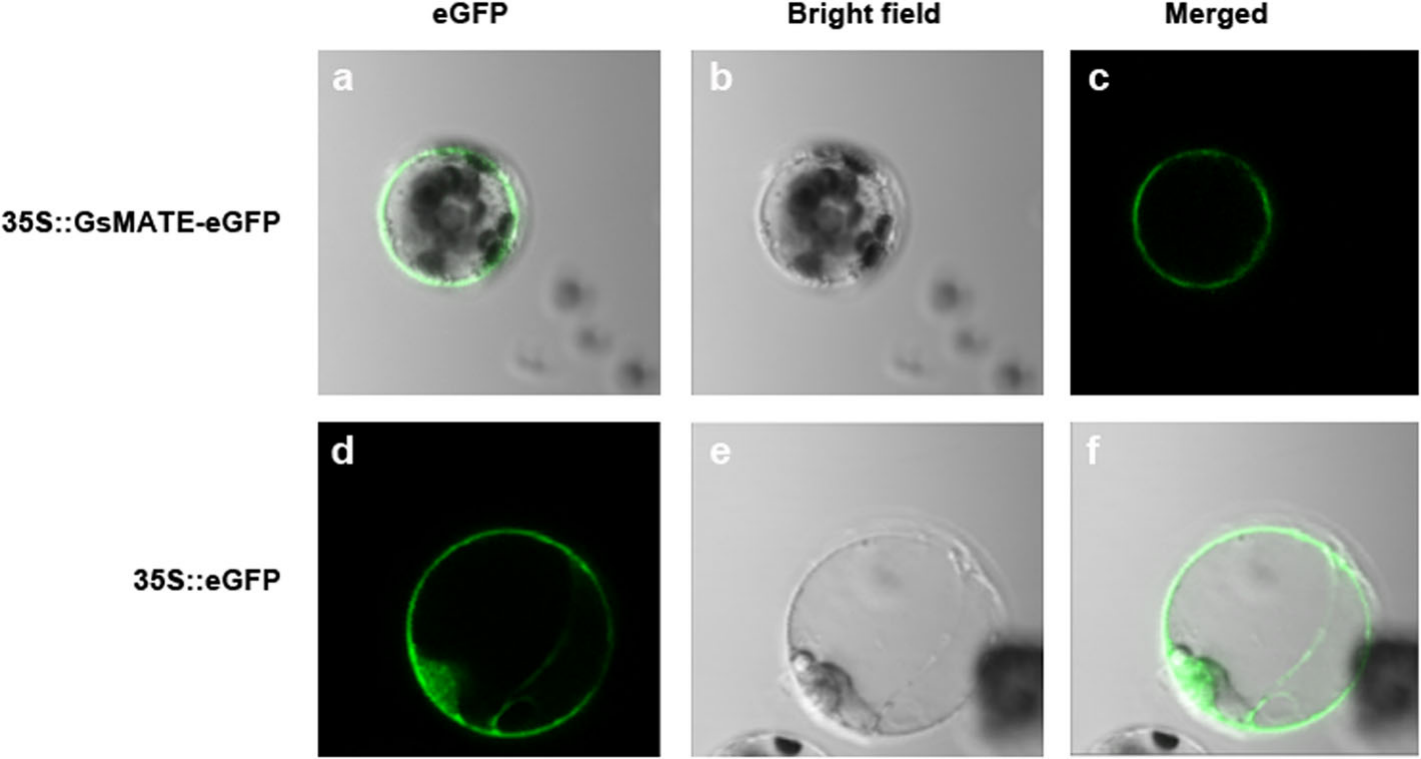
Analysis of GsMATE protein localization. **a** Localization of GsMATE-GFP fusion protein or GFP alone in *Arabidopsis* protoplasts; **b** and **e** Corresponding bright-field images; **c** and **f**. Merged images; **d** pYL322-d1-eGFP vector. The GsMATE-GFP fusion vector was constructed by cloning the full coding sequence of GsMATE (without TAA) into the *Bam*HI and *Kpn*I sites of pYL322-d1-eGFP vector. *Arabidopsis* protoplasts were transformed using the heat-shock method. After 24 h, GFP or GsMATE-GFP fusion protein expression (driven by the CaMV 35S promoter) was visualized by fluorescence microscopy

To examine its subcellular localization, the *GsMATE* sequence was fused to the GFP reporter gene at the 5′-terminus under the control of the CaMV 35S promoter. The recombinant constructs encoding the GsMATE-GFP fusion protein and GFP alone (pYL322-d1-eGFP vector) were then transformed into *Arabidopsis* protoplasts by heat-shock. As shown in Fig. 4a, the GsMATE-GFP fusion protein accumulated mainly in the membrane with strong signals of green fluorescence located in the cell membrane. In contrast, GFP alone was observed as green fluorescence emitted throughout the whole cell (Fig. 4d). These observations were consistent with the predicted function of GsMATE as a membrane protein (Additional file 3: Figure S1b).

### Generation and molecular identification of GsMATE transgenic lines

More than 19 *GsMATE* transgenic plants of the T_1_ generation were further identified by PCR amplification of the hygromycin gene encoded by the pCAMBIA1301 vector (data not shown). The results indicated that *GsMATE* has been integrated into the *Arabidopsis thaliana* genome (Additional file 4: Figure S2a). Transgenic lines of the T_2_ generation were identified by qRT-PCR analysis of the *GsMATE* overexpression in *Arabidopsis* at the RNA level. Two transgenic *GsMATE* lines were selected to investigate the phenotype and mechanism of resistance to acidic aluminum (Additional file 4: Figure S2b).

### GsMATE enhanced the tolerance of Arabidopsis to Al toxicity

**Fig. 5:**
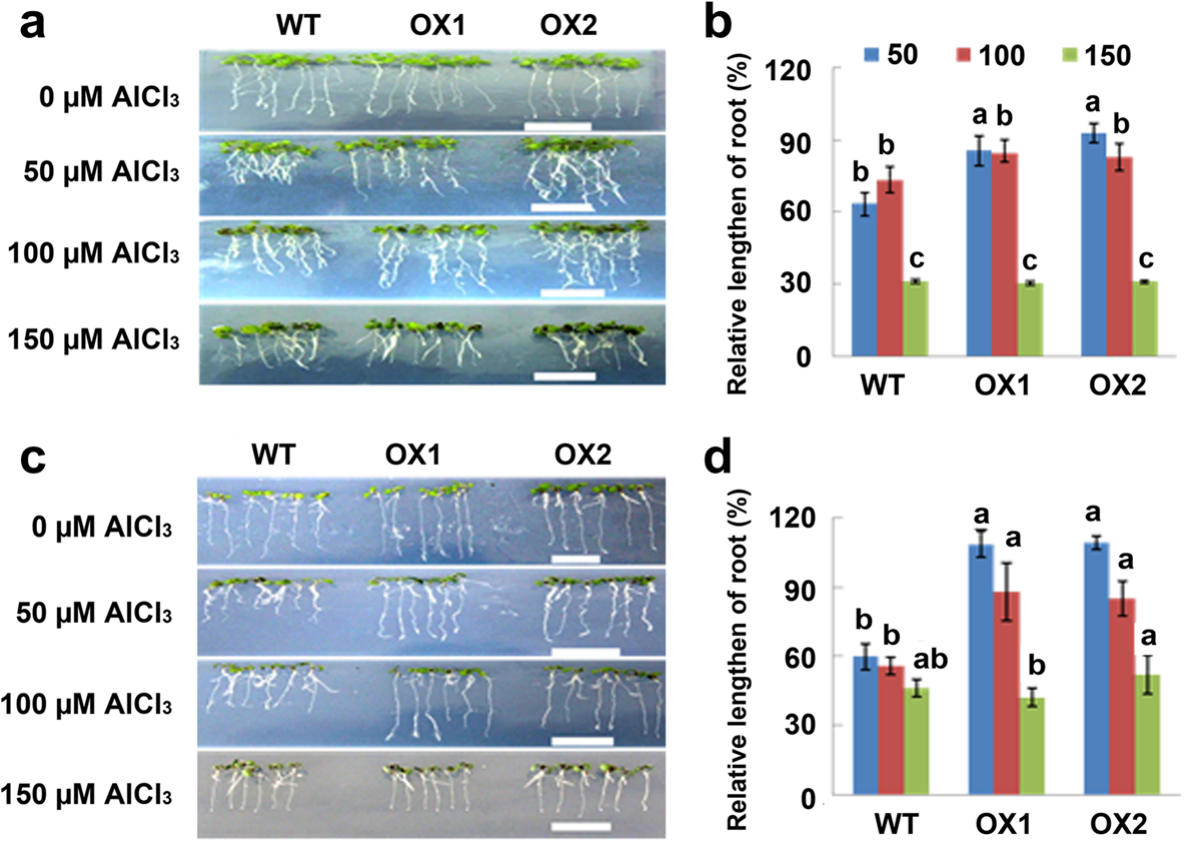
*GsMATE* increases Al tolerance of transgenic Arabidopsis lines. **a** Al tolerance phenotypes of *GsMATE* in transgenic lines*.* **b** Statistical analysis of relative root length. WT: wild-type of Arabidopsis (Col-0); OX1/OX2: *GsMATE* overexpression transgenic lines. Two days after germination, seedlings were transferred to culture medium containing 0, 50, 100, and 150 μM AlCl_3_ (pH 4.3, 0.5 mM CaCl_2_). After 7 days in culture, images of the phenotypes of the *GsMATE* transgenic lines were recorded for statistical analysis. Data were represented as the mean ± SE of three biological replicates (t-test, *p* = 0.05). Data were measured using Image J software, analyzed using SPSS20, and plotted using EXCEL2000

To investigate the responses of *GsMATE* transgenic lines to Al stress, *Arabidopsis* seedlings were exposed to AlCl_3_ at 0, 50, 100, and 150 μM for 7 days. The results showed that the relative root length (taproots and lateral roots) of *GsMATE* transgenic lines was significantly greater (>80%) than those of the wild-type under Al stress at 50 and 100 μM AlCl_3_ (Fig. 5a). However, under 150 μM AlCl_3_, root elongation was completely inhibited in both the *GsMATE* transgenic lines and wild-type *Arabidopsis* performing similar phenotypes and relative root lengths (Fig. 5a). Furthermore, the *Arabidopsis* seedlings exhibited similar Al-resistance phenotypes under Al stress for 2 days (Additional file 5: Figure S3). These results indicated that *GsMATE* overexpression may improve the tolerance to aluminum stress in *Arabidopsis*.

To further confirm the involvement of *GsMATE* in resistance to aluminum toxicity, T_3_ generation *Arabidopsis* seedlings were harvested after developing for 12 days in 1/2 MS media, followed by treatment with AlCl_3_ (0, 50, 100, and 150 μM) for 30 min. Hematoxylin staining of the roots of *GsMATE* transgenic lines increased in intensity with the increased concentration of aluminum. Furthermore, at the same concentration of AlCl_3_, the intensity of hematoxylin staining was much greater in the roots of wild-type *Arabidopsis* seedlings than those of the *GsMATE* transgenic plants (Additional file 5: Figure S3). These observations indicated that *GsMATE* may enhance the resistance of transgenic plants to Al toxicity by inhibiting the accumulation of aluminum in the roots.

## Discussion

The present study aimed to investigate the function of *GsMATE* gene response to acidic aluminum. Previous reports showed that MATE proteins represented a large family in bacteria, fungi, plants and mammals which can transport numerous substrates [14]. The plant MATE proteins characterized to transport citrate are involved in several physiological processes including Al-tolerance [1, 6, 15–19, 21–24, 26], iron translocation [23, 30, 32], heavy metals [33], toxins [34], vacuolar transport of nicotine [34–37], chloride channels [38]; ABA efflux [39]; transport of secondary metabolites such as alkaloids, flavonoids, and anthocyanins [36, 40–42]; and phosphorus efficiency. In this study, a *GsMATE* gene encoding an Al-activated citrate MATE transporter was cloned from the BW69 line of wild soybean and used to characterize the functional properties as well as its potential role in plants. Our results have shown that GsMATE plays a role in citrate secretion. First, *GsMATE* is expressed mainly in roots with an expression pattern that is specifically up-regulated by Al with higher expression level in the root tips (Figs. 1 and 2). Second, similar to other MATE transporters involved in Al-induced citrate secretion, the GsMATE protein is also localized predominantly at the plasma membrane in protoplasts (Fig. 4).

The soybean MATE family is large, consisting of at least 117 members located on chromosomes 1 to 20 with uneven distribution. Most MATE genes exhibit tissue-specific expression patterns [54]; however, genome-wide association analysis of MATE transporters showed that soybean MATE family could be classified into four subfamilies comprising a total of ten smaller subgroups with diverse potential functions, including extrusion of compounds, regulation of disease resistance, transport and accumulation of flavonoids or alkaloids, and responses to abiotic stresses [54]. Based on previously reported MATE proteins, further analysis revealed that eight soybean MATE transporters clustered together were related to Al detoxification and iron translocation [54]. In the present study, the phylogenetic analysis was carried out using the available amino acid sequences of reported MATE proteins and the eight soybean MATE transporters. Homology analysis showed that GsMATE which was clustered with eight Al-induced soybean MATE transporters in Group III covering Al detoxification and iron translocation (Fig. 1). Amino acid sequence alignment showed that GsMATE has only one different amino acid at the end of C-terminal compared to that of GmMATE47 (data not shown). In addition, GmMATE87 and GmFRD3a have the same sequence, while GmMATE47 has the same sequence as GmFRD3b [31] with the exception of 15 additional amino acids at the end of N-terminal of GmMATE47 (Fig. 1, data not shown). Previous studies showed that the expression of GmFRD3a and GmFRD3b induced by iron deficiency in the iron-efficient reference cultivar Williams 82 played a role in iron translocation in soybean [31]. Differential gene expression analysis indicated that *GmMATE47* and *GmMATE87* were related to aluminum detoxification and iron translocation. While *GmMATE75*, which is the candidate gene among the eight identified MATE genes for Al tolerance in soybean, is rapidly up-regulated by Al stress [54]. In our study, heterogenous overexpression of *GsMATE* enhanced the resistance to Al stress in *Arabidopsis* (Fig. 5). Therefore, our results provided a foundation for further investigation of the functions of soybean MATE genes including the candidate gene for Al tolerance in soybean.

## Conclusions

The *GsMATE* gene encodes a transmembrane protein that is enriched in soybean roots, and is up-regulated in response to acidic aluminum. *GsMATE* overexpression enhances the resistance to Al toxicity in transgenic *Arabidopsis* plants. These results indicated that the GsMATE protein may be responsible for external detoxification of Al by mediating root citrate efflux.

## Additional files

Additional file 1: List of primers. (DOCX 18 kb)

Additional file 2: CDS sequencing information of *GsMATE*. (DOCX 16 kb)

Additional file 3 Putative domains and TMs of GsMATE protein. (DOCX 702 kb)

Additional file 4: Molecular identification of GsMATE transgenic Arabidopsis lines. (DOCX 272 kb)

Additional file 5: Identification of GsMATE transgenic Arabidopsis lines by hematoxylin staining. (DOCX 119 kb)

## Abbreviations

ABA: Abscisic acid
Al: Aluminum
BW69 and JW81: two lines of *Glycine soja*
CDS: Coding sequence
Gs: *Glycine soja*
MATE: Multidrug and toxic compound extrusion
NCBI: the National Center for Biotechnology Information
ORF: Open reading frame
OX1/OX2: Overexpression transgenic lines of *GsMATE*
WT: Wide type of Arabidopsis (Col-0)
