# Supplementary material for: GsMATE encoding a multidrug and toxic compound extrusion transporter enhances aluminum tolerance in Arabidopsis thaliana
Source: BMC Plant Biol. 2018 Sep 29;18:212. doi: 10.1186/s12870-018-1397-z (PMC6162897; doi:10.1186/s12870-018-1397-z)
Supplement: Supplementary file 1 — List of primers. (DOCX 18 kb) [file 12870_2018_1397_MOESM1_ESM.docx]

## **Table S1 List of primers**

| Serial number | Primer name | Primer sequence | Purpose |
| --- | --- | --- | --- |
| 1 | GmMATEL-1 | 5´ATGTTTATCTTTCGTGC3´ | *GsMATE* cloning |
| 2 | GmMATEL-2 | 5´ACCCACAAAGGGAGCA3´ |  |
| 3 | pZeroback/Blunt-F | 5'CGACTCACTATAGGGAGAGCGGC3' | GsMATE-pZeroback |
| 4 | pZeroback/Blunt-R | 5'AAGAACATCGATTTTCCATGGCAG3' |  |
| 5 | MATELQ-1 | 5'CGGGATCCATGATGCCCCTTTTGATGTTAT3' | GsMATE-pCAMBIA1301 |
| 6 | MATELQ-2 | 5' GGGGTACCCTAAAGCCCAACATTGTTTACC3' |  |
| 7 | ACT3-F | 5'GCACCACCGGAGAGAAAATA3' | qRT-PCR |
| 8 | ACT3-R | 5'GTGCACAATTGATGGACCAG3' |  |
| 9 | GsMATE-F | 5' GGCATTGATGGGTTGAGTGG3' |  |
| 10 | GsMATE-R | 5' GGCTTGATTCTTGGGCTGG3' |  |
| 11 | GMATES-1 | 5'GGGGTACCATGATGCCCCTTTTGATGTTAT3' | GsMATE-pYL322-d1 |
| 12 | GMATES-2 | 5'CGGGATCCAAAGCCCAACATTGTTTACCTT3' |  |
| 13 | hpt-F | 5' ACTTCTACACAGCCATCGGTCC3' | Molecular identification for transgenic lines by *Hygromycin* gene |
| 14 | hpt-R | 5' AGCGAGAGCCTGACCTATTGC3' |  |
| 15 | At-TUB-1 | 5'ATCGATTCCGTTCTCGATGT3' | *Tubulin* for qRT-PCR |
| 16 | At-TUB-2 | 5'ATCCAGTTCCTCCTCCCAAC3' |  |

All the primers were used for gene cloning, vector construction, molecular identification, and expression analysis.
