# Supplementary material for: GsMATE encoding a multidrug and toxic compound extrusion transporter enhances aluminum tolerance in Arabidopsis thaliana
Source: BMC Plant Biol. 2018 Sep 29;18:212. doi: 10.1186/s12870-018-1397-z (PMC6162897; doi:10.1186/s12870-018-1397-z)
Supplement: Supplementary file 2 — CDS sequencing information of GsMATE. (DOCX 16 kb) [file 12870_2018_1397_MOESM2_ESM.docx]

**CDS sequencing information of *GsMATE***

ATGATGCCCCTTTTGATGTTATTTTCTGGCATAAGGAATGCTTTCTCAAGTGATGAATTAGGCTTGGAAATACTGAATATTGCACTCCCTACTACTCTGGCTTTGGCAGCAGATCCTATTGCTTCTCTAATTGATACTGCATTCATTGGCCACATAGGTCCTGTAGAGCTAGCTGCTGTGGGAGTCTCAATTGCTATTTTCAATCAAATCTCAAAAATTACAATAATTCCACTAGTCAGTGTCACAACCTCTTTGGTTGCCGAGGAAGATGCTGCTGATGAACAAAATCAACAGTCAGAAAAAGAAATGCTGATGAAGGTCTCTAATGAGGATGTAAAATTGGATGTACATGACCATATAGAGAAAGCTGGTAACTCTTCATCAGCAAATGTTGGTAGAGTAGCTAAACTTAAACATGACAAAAGCTATATTCCATCAGCATCATCAGGAGTAGTTATTGGTGGTGTGCTTGGGGTCCTACAAGCTCTCTTTCTCATTTTTACAGCTAAACCAATGTTGAGTTACATGGGCGTTGATTCGAATTCTCCTATGTTTAAACCAGCACAACAATACTTGACATTGAGGTCATTTGGTGCACCAGCAGTTATTATTTCTATGGCAATTCAAGGAGTTTTTCGTGGAATCAAAGATACAAAAACTCCTTTATATGCTACAGTAATGGGAGATGTAACAAATATTATTTTAGATCCGTTACTTATGTTCGTACTGCGTTTGGGAGTCAATGGGGCAGCCATTTCCCACATTATCTCCCAGTACTTGATTTCCATAATGCTACTGTGGAGTTTAATGCAACAAGTTGTTCTTATTCCTCCAAGTATCCAAGACTTTCAATTTGGGAAGATTCTTAAAAATGGGTTTCTATTATTGATTAAAGTTGCATCTGTGACTTTCTGTGTGACCTTGTCAGCATCCCTAGCAGCAAGGAAAGGATCAACAACAATGGCTGCATTTCAAATCTGCTTACAGATTTGGATGGCAACCTCTTTGCTTGCTGATGGATTGGCTGTTGCGGGACAAGCTATTATTGCAAGTGCATTTGCGAGAAATGATTACAAAAGGGTTATCGCATCTGCCTCACGTGTGCTGCAGCTTGGCTTGATTCTTGGGCTGGTGCTCTCTGTCCTTCTTTTAAGTCTACTACCATTTGCTTCTAGGTTATTTACTAATGACAACAATGTTCTGCAACTTATCAGTATTGGCATTCCATATGTTGCTGCCACTCAACCCATCAATGCCCTGGCATTTGTTTTCGATGGAGTCAACTATGGAGCTTCAGATTTCACATATTCTGCATACTCAATGATTATGGTAGCATTGGTGAGCATATTGAGTTTATATACGCTGTCCTCAAGCCTTGGCTTTACCGGTATCTGGATTGCGTTGTCGATTTACATGACTCTAAGGATATTTGCAGGCTTTTGGAGGATTGGTACTGGATCAGGGCCTTGGAGCTTCCTTCAGGTAAACAATGTTGGGCTTTAG

Coding sequence (CDS) of *GsMATE* is 1503 bp which was cloned with the probe sequence of BM732932.1 using BW69 line of *Glycine soja* and sequenced by Sangon Biotech.

Table S2 The probe information of gene expression profiles from BW69 line of *Glycine soja*

| **Probe annotation** |  |  |  | **Test** | **Control** | **Difference analysis** |  |
| --- | --- | --- | --- | --- | --- | --- | --- |
| ProbeID | TargetID | Accessions | Description | AL-G | CK-G | log2 AL-G VS CK-G | Regulation |
| BM732932.1_486 | BM732932.1 | gb\|BM732932.1 | Glycine max cDNA clone SOYBEAN CLONE ID: Gm-c1063-3264 5 similar to TR:Q9SYD6 Q9SYD6 F11M15.20 PROTEIN, mRNA sequence | 10.649 | 9.47 | 1.179 | upregulation |

Gene expression profiles of resistant to acid aluminum using BW69 line of *Glycine soja* (data unpublished). The full probe of BM732932.1 was used to find the full sequence of *GsMATE* from the National Center for Biotechnology Information. AL-G: soybean seedlings was treated under the solution of 50 μM AlCl_3_ (pH 4.3, 0.5 mM CaCl_2_). CK-G: soybean seedlings was treated under the solution of 0 μM AlCl_3_ (pH 4.3, 0.5 mM CaCl_2_).
