# Supplementary material for: GsMATE encoding a multidrug and toxic compound extrusion transporter enhances aluminum tolerance in Arabidopsis thaliana
Source: BMC Plant Biol. 2018 Sep 29;18:212. doi: 10.1186/s12870-018-1397-z (PMC6162897; doi:10.1186/s12870-018-1397-z)
Supplement: Supplementary file 3 — Putative domains and TMs of GsMATE protein. (DOCX 702 kb) [file 12870_2018_1397_MOESM3_ESM.docx]

**
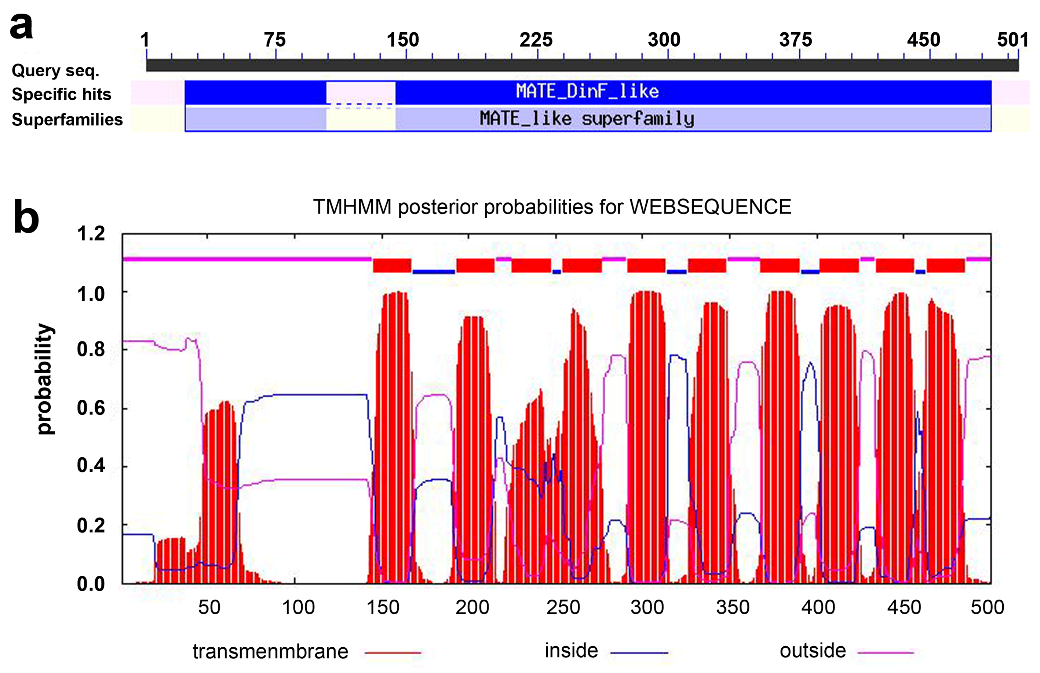
**

**Fig.S1 Putative domains and TMs of GsMATE protein**

## **a**. Putative domains of GsMATE. **b**. TMHMM posterior probabilities of GsMATE. 501 [amino-acid residue](http://www.baidu.com/link?url=IT1-ZystCFzUfPYlD_BTVG1hQ9qN0TGor6E5pTv3eo4RwRv0Z4LDQ4YOy2tNp1OhTc3f4qNCmc-Rp3ZRbux0a-KJcmlwpHAHmfXXI-QIei-o7k8o6IQwNivpld1JjdVa)s were used to detect the conserved domains of GsMATE with the website of protein blast: <https://blast.ncbi.nlm.nih.gov/Blast.cgi,> and predict transmembrane helices of GsMATE with the website of TMHMM Server v. 2.0: <http://www.cbs.dtu.dk/services>/TMHMM/.
