# Supplementary material for: GsMATE encoding a multidrug and toxic compound extrusion transporter enhances aluminum tolerance in Arabidopsis thaliana
Source: BMC Plant Biol. 2018 Sep 29;18:212. doi: 10.1186/s12870-018-1397-z (PMC6162897; doi:10.1186/s12870-018-1397-z)
Supplement: Supplementary file 4 — Molecular identification of GsMATE transgenic Arabidopsis lines. (DOCX 272 kb) [file 12870_2018_1397_MOESM4_ESM.docx]

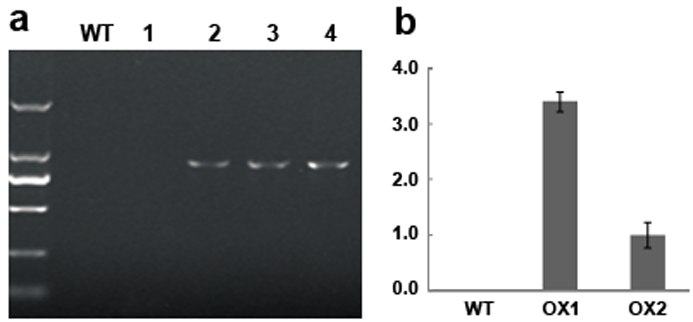


**Fig.S2 Molecular identification of *GsMATE* transgenic *Arabidopsis* lines**

**a**. PCR detected *GsMATE* transgenic lines in Arabidopsis. **b.** Expression analysis of *GsMATE* in transgenic lines of T_3_ generation. WT: wide type (Col-0); 1: PCR reaction solution with H_2_O as template; 2-4: *GsMATE* transgenic plants from different lines; OX1/OX2: overexpression transgenic lines of *GsMATE*. PCR measurement of transgenic Arabidopsis was carried out by the method of *transDirect*^TM^ Plant Tissue PCR Kit. Data represents three biological replications, and error bars represent SE. The specific primers of PCR and qRT-PCR for molecular identification of *GsMATE* transgenic Arabidopsis lines were listed in Additional file 1. The methods for PCR and qRT-PCR were performed with the programs described previously in detail [57, 58].
