# Supplementary material for: GsMATE encoding a multidrug and toxic compound extrusion transporter enhances aluminum tolerance in Arabidopsis thaliana
Source: BMC Plant Biol. 2018 Sep 29;18:212. doi: 10.1186/s12870-018-1397-z (PMC6162897; doi:10.1186/s12870-018-1397-z)
Supplement: Supplementary file 5 — Identification of GsMATE transgenic Arabidopsis lines by hematoxylin staining. (DOCX 119 kb) [file 12870_2018_1397_MOESM5_ESM.docx]

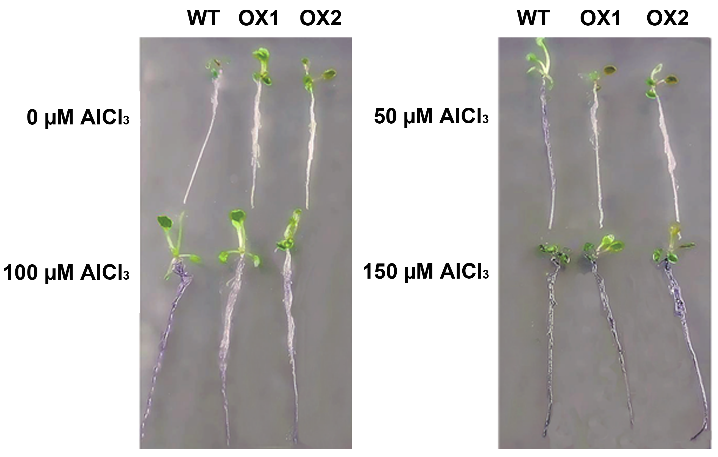


**Fig.S3 Identification of *GsMATE* transgenic *Arabidopsis* lines by the method of Hematoxylin staining**

Arabidopsis seeds of wild type and *GsMATE* transgenic lines of T_3_ generation were treated at 4℃ for four days after sowning on 1/2 MS Phytagel medium (pH 5.8), and then transferred to light culture chamber to culture vertically for 12 days. The seedlings were selected to grow for six hours in the simple calcium solutions containing 0, 50,100,150 µM AlCl_3_, respectively. Ten seedlings from each treatment were dyed by Hematoxylin solution. WT: wide type (Col-0); OX1/OX2: overexpression transgenic lines of *GsMATE*.
